# Notes and News

**Published:** 1891-02-14

**Authors:** 


### Notes and News.

Collected and Communicated.

Both the North-West London and the Metropolitan Hospitals have issued appeals for additional funds. The Committee of the former institution owe more than £2,000, towards meeting which they have only about £700; while the latter needs £1,300 to pay off the Christmas bills and help on the funds for the current year.

The annual meeting of subscribers to the Canterbury Dispensary was held under the presidency of the Mayor, W. W. Mason, Esq. The Committee presented their fifty-fifth annual report. During the past year the number who have applied for and received relief far exceeded the average number for former years, which increase was probably caused by a severe epidemic of influenza in the winter, and an epidemic of measles in the spring. At such times the calls on the institution are very great, and it is difficult to realise what the poor would do in time of sickness without this charity. The financial statement showed invested capital amounting to £4,598 17s. 7d.

A public meeting was held in Birmingham to promote a scheme for rebuilding the General Hospital on a new site, the old building having become insanitary in its surroundings, and inadequate to the calls upon it. The late Miss Ryland left £25,000 for this project, the total sum required being £115,000, deducting the sum realizable from the sale of the old site. The public of the city and neighbourhood were asked to find about £70,000. Seldom has an appeal received a more liberal response. No less a sum than £75,000 has already been raised, and this has been effected by the subscriptions of only 320 persons. The new hospital will be erected on a site between Steelhouse Lane and St. Mary's Square.

One of the chief evidences of the useful work the City of London Truss Society is doing is the number of working men's societies which have joined the association. No less than 104 distinct societies of this kind subscribe to the charity, showing clearly how valuable its work appears in the eyes of the poor. The past year has been a most favourable one for the society, for the subscriptions and collections have been larger than usual, and though the amount actually received from legacies has fallen off, yet notice has been received of a large legacy left by the late Sir John Tyler, which does not appear in the 1890 accounts. Despite this, however, the increasing demands on the charity have rendered it necessary to sell out £2,000 of stock, a resolution carrying this into effect being passed at a special general meeting, held after the annual meeting of Governors on the 4th inst., Mr. John Norbury, Treasurer, in the chair. Nor is this to be wondered at when we read in the report for the year 1890 that only one letter is ever required for the relief of a sufferer from hernia, however aggravated his or her case may be; and it is often found that upon that one letter nearly the whole value of a guinea subscription—for which four letters are issued—is given by the society. Naturally the progress of time is telling heavily on the old and early supporters of the charity, and though still vigorous in its work, yet year by year it loses more and more of those who watched its earlier history and aided in its progress. Unless, therefore, new friends come forward to stand in the place of old ones, the subscription list must diminish in numbers, and the funds must decrease in amount, a climax which, it is earnestly to be hoped, will never occur.

The report for 1890 of the Glasgow Royal Infirmary contains an interesting and important proposal. This is, that the directors should take over, together with its assets, which amount to over £10,000, the ophthalmic institution, which has been carried on as a separate hospital for diseases of the eye for about 20 years. The institution is to be removed to Castle Street, and form a department of the Infirmary which has long been urgently needed. Subjoined to the report is an appeal for funds to form an efficient endowment for the Medical School of St. Mungo's College. The Infirmary depends upon this College for a sufficient supply of dressers, without which the hospital could not maintain the high standard of excellence with which it at present performs its work. The appeal therefore is deserving of favourable consideration by all well-wishers of the Royal Infirmary.

Baron Von Schröder presided at the annual court of Governors of the German Hospital, which was held at the Cannon Street Hotel on January 30th. After the reading and adoption of a satisfactory report and balance-sheet, Mr. A. T. Allen moved that Rule 15 be altered, so that the senior hon. physician and the senior hon. surgeon be *ex-officio* members of the Committee, and said that the Committee very much regretted that Drs. Lichtenberg and Tuchmann, who had rendered such splendid services to the hospital, had thought proper to oppose this step. Dr. Lichtenberg moved as an amendment, that a full physician and a full surgeon be added to the Committee, to be elected according to seniority every three years, and said that by the scheme of the Committee much extra work would be thrown on the medical officers elected upon the Committee. If the proposal became law, he, as Senior Surgeon, would be on the Committee, and would have to attend the Board meetings. Dr. Tuchmann, in seconding the amendment, complained that the Committee had acted inconsiderately towards the honorary medical staff. After some discussion, the amendment was negatived. Dr. Lichtenberg then announced that the connection of himself and Dr. Tuchmann with the hospital must be considered at an end. A vote of thanks to the Chairman having been passed, the meeting terminated.

The quarterly meeting of the Governors of the Norfolk and Norwich Hospital was held on the 10th inst., H. W. B. Edwards, Esq., in the chair. Mr. Collins, the Secretary, read the report, and asked on behalf of the Board for permission to expend a sum not exceeding £75 for the purpose of heating the Isolation Block with hot water pipes. Sir Peter Eade, in moving the adoption of the report, remarked that why hot water pipes were not carried to the Isolation Block when put into the Hospital he was unable to say. The block was not in constant use like the other wards, but it ought to be always ready for emergencies, so that any patient could be moved to it at five minutes' notice, if found desirable. The report was adopted, and the meeting then discussed the report of the committee, which had been appointed to consider the question of taking over the "Shrubbery" house and converting it into a nurses' home and pay hospital. The Committee were unanimous in desiring to see in connection with the Hospital a central nurses' home from which the nursing wants of city and county could be provided, and also that the home should be adapted for receiving and nursing patients who are able to pay for same. The Committee recommended that the taking over of the Shrubbery be postponed for a time. They further recommended that as many additional probationers or nurses as can be accommodated in the Hospital be engaged as the commencement of a nurses' home.

